# Mixed effect of increasing outflow of medical patients from an emergency department

**DOI:** 10.1186/s13584-021-00491-9

**Published:** 2021-10-27

**Authors:** Joseph Mendlovic, Todd Zalut, Gabriel Munter, Ofer Merin, Amos M. Yinnon, David E. Katz

**Affiliations:** 1 Shaare Zedek Medical Center, P.O. Box 3235, 91031 Jerusalem, Israel; 2 Division of Internal Medicine, Shaare Zedek Medical Center, Jerusalem, Israel; 3 Hadassah Faculty of Medicine, Hebrew University, Jerusalem, Israel

**Keywords:** Reimbursement, Emergency department, Medical patients, Medical departments, Early morning discharge, Length of stay

## Abstract

**Background and aim:**

Since 2014, the annual number of patients entering our emergency department (ED) has increased significantly. These were primarily Internal Medicine (IM) patients, and of these, 25–30% were admitted. The present governmental policy presents a deterrent to adding IM beds for these patients, and Emergency and IM departments cope with ever-increasing number of IM patients. We describe a quality improvement intervention to increase outflow of IM patients from the ED to the IM departments.

**Methods:**

We conducted a quality improvement intervention at the Shaare Zedek Medical Center from 2014 to 2018. The first stage consisted of an effort to increase morning discharges from the IM departments. The second stage consisted of establishing a process to increase the number of admissions to the IM departments from the ED.

**Results:**

Implementation of the first stage led to an increased morning discharge rate from a baseline of 2–4 to 18%. The second stage led to an immediate mean (± SD) morning transfer of 35 ± 7 patients to the medical departments (8–12 per department), providing significant relief for the ED. However, the additional workload for the IM departments’ medical and nursing staff led to a rapid decrease in morning discharges, returning to pre-intervention rates. Throughout the period of the new throughput intervention, morning admissions increased from 30 to > 70%, and were sustained. The number of patients in each department increased from 36 to 38 to a new steady state of 42–44, included constant hallway housing, and often midday peaks of 48–50 patients. Mean length of stay did not change. IM physician and nurse dissatisfaction led to increased number of patients being admitted during the evening and night hours and fewer during the morning.

**Conclusion:**

We describe a quality improvement intervention to improve outflow of medical patients from the ED in the morning hours. The new ED practices had mixed effects. They led to less ED crowding in the morning hours but increased dissatisfaction among the IM department medical and nursing staff due to an increased number of admissions in a limited number of hours. The present governmental reimbursement policy needs to address hospital overcrowding as it relates to limited community healthcare beds and an aging population.

## Introduction

All hospitals and emergency departments need to cope with a reasonable mismatch between bed-availability and patient needs. A small surplus of hospital beds allows for accommodation of seasonal upsurge in demand but carries the price of lost revenue. In order to induce hospitals to curb duration of medical admissions, the Israeli Ministry of Health (MOH) publishes data on patient length of stay (LOS) for Israeli hospitals. This data is not controlled for variables such as age, diagnosis, and functional status [1, 2]. In addition, the MOH financially penalizes hospitals that increase bed numbers in relationship to the national population growth rate, without factoring in the growth in the significantly increasing elderly population and the rising burden of chronic disease. The result of this policy has been a > 100% bed occupancy rate almost nationwide in internal medicine (IM) departments, overcrowding of emergency departments (ED), and a decreased incentive to build additional departments or add beds. While this is a nationwide problem, the capacity strain at the Shaare Zedek Medical Center (SZMC) has been exceptional [3–8].

As a response to the public outcry over the crowded EDs in all of the 26 Israeli general hospitals, the MOH hired a consultant agency. After making observations in our ED and analyzing occupancy and admission data, the consultants recommended that there be an increase in patients admitted by 11:00 am, as this coincided with peak ED occupancy. This led the hospital’s management to experiment with a quality improvement (QI) intervention, i.e., transferring a large number of patients from the ED to the IM departments by 11:00 am according to a specified formula without regard for bed availability in the departments. It is the purpose of this paper to describe this intervention and its effect on patient flow at our medical center.

## Methods

This QI intervention study was carried out from 2014 to 2018 at SZMC, a university affiliated general hospital in Jerusalem, Israel. This 1000-bed hospital provides services for all specialties, with the exception of solid organ transplantation and radiation oncology. The general ED has 45 beds with a surge capacity up to 120 beds, and services 90,000 patient visits per year (pre-Covid-19). The IM division consists of three general medical departments (A, B, and C), each staffed to take care of 36 patients, a smaller 22-bed medical department (D) for short admissions originally situated next to the ED, and a 39-bed acute geriatric department [9]. During the study period, only a marginal number of beds (less than 5) were added to the IM division, which were not expected to have a significant influence on the outcomes of the new intervention. In addition, there is a medical "observation" unit in the ED taking care of the overflow of medical patients for whom no bed is available, mainly consisting of high-functioning patients with expected admissions spanning less than 48 h. Oncology and Cardiology are separate departments, not part of the IM department. The data analyzed and collected included demographic data of patients entering the ED and being admitted to the IM departments over 5 years, starting 2 years prior to implementation (2014–2015) of the new admission intervention and subsequently throughout its 3-year course (2016–2018). Admissions to the acute geriatric department were excluded from the study, since it was not being managed under the intervention.

The development of this intervention consisted of two stages. Stage one was initiated in June 2015. This stage involved a shift in workflow of the IM departments to attempt to increase their morning discharge rate prior to 11:00 am from 5 to 20%. This increase would enable the ED to transfer between 15 and 20 patients every morning to the IM departments. If this could not be achieved, then stage two of the QI intervention would be initiated. This would involve admitting half of the patients waiting in the ED for admission by 11:00 am to the 4 medical departments.)The other half would remain in the ED to be taken care of by the medical staff of the ED itself). The number of patients to be admitted by 11:00 am was derived from an analysis performed by the MOH consultant, coinciding with the average number of patients present in the ED needing IM admission during the morning hours.

## Results

**Fig. 1:**
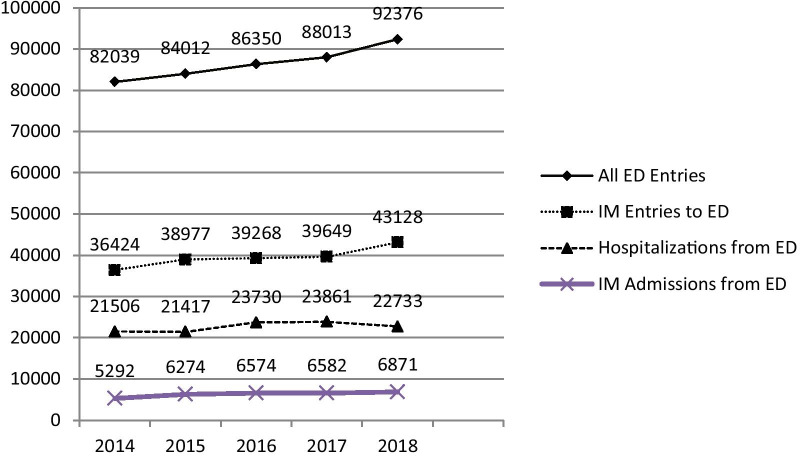
ED and IM admissions, 2014–2018. *ED* emergency department, *IM* internal medicine departments

Between 2014 and 2018, the number of patients entering the ED at SZMC increased by 13% (i.e., more than 10,000 patient visits over this time period). Of these, the most significant increase was that of IM patients (Fig. 1). Approximately 25–30% of all IM patients seen in the ED are admitted to the IM Division. This does not include patients admitted to geriatrics, cardiology, or hematology-oncology. Demographic data of the patients admitted to the IM departments are shown in Table 1. Although the majority of patients arrive from the ED, an additional 10–20% of patients were transferred from other departments. Over the timeline of this study, there remained a significant similarity of patients admitted to IM departments A and B, as indicated by comparable mean age, median and mean duration of stay, as well as mortality rates. IM department C moved from its original location next to the ED to its current site next to IM department A and B on the eighth floor at the end of 2017, upon which its patient demographics changed rapidly; specifically, the mean age increased from 68 to 77 years, median length of stay from 3 to 4 days, and mortality from 3.7 to 8.5%, nearly closing the gap with IM departments A and B. IM department D, consisting of 22 beds, was located in a small hospital in down-town Jerusalem. IM department D was closed at the end of 2017 and reopened in the space vacated by IM department C. For IM department D, the mean age of its patients in 2018 was younger than the other medical departments, median LOS was 3 days, and they had a 3.3% mortality rate. While containing about half as many beds as the other medical departments, IM department D’s number of annual admissions equaled that of the other departments due to a rapid turnover of patients.

**Table 1:** Demographic data of patients admitted to IM departments, 2014–2018

	2014	2015	2016	2017	2018
Number of beds					
Medicine A	36	36	36	36	36
Medicine B	36	36	36	36	36
Medicine C	36	36	36	36	36
Medicine D	22	22	22	22	22
Mean admissions from ED					
Medicine A	1587	1340	1621	1723	1553
Medicine B	1368	1278	1509	1601	1546
Medicine C	2454	2353	2242	2298	2005
Medicine D	1312	1303	1202	960	1767
Total admisssions^a^					
Medicine A	1774	1546	1867	1971	1784
Medicine B	1720	1612	1836	1939	1879
Medicine C	2696	2651	2543	2563	2182
Medicine D	1336	1320	1218	992	1880
Mean age					
Medicine A	77	77	77	78	78
Medicine B	76	75	75	77	76
Medicine C	68	72	73	74	77
Medicine D	75	74	74	75	68
Median LOS					
Medicine A	5	6	5	5	5
Medicine B	5	5	5	4	5
Medicine C	3	3	4	4	4
Medicine D	5	5	4	4	3
Mean LOS (SD)					
Medicine A	7.5 (8.7)	8.6 (11)	7.7 (11.8)	7.5 (10.1)	8.5 (12.5)
Medicine B	7.8 (10.6)	8.3 (10.8)	7.8 (10.9)	7.7 (10.5)	8.2 (11.4)
Medicine C	4.9 (5.5)	5.1 (6.1)	5.6 (7.9)	6.1 (8.7)	7.0 (10.6)
Medicine D	6.2 (5.9)	6.1 (9.4)	6.1 (7.3)	6.3 (24)	4.3 (5.2)
Deaths, n (%)					
Medicine A	277 (15.6)	230 (14.9)	221 (11.8)	211 (10.7)	205 (11.5)
Medicine B	242 (14.1)	214 (13.3)	203 (11.1)	244 (12.6)	232 (12.3)
Medicine C	101 (3.7)	171 (6.4)	154 (6.1)	184 (7.2)	185 (8.5)
Medicine D	59 (0.5)	58 (4.4)	33 (2.7)	25 (2.5)	63 (3.3)

*IM* internal medicine, *ED* emergency department, *LOS* length of stay, *SD* standard deviation

^a^Including transfers from other departments

**Fig. 2:**
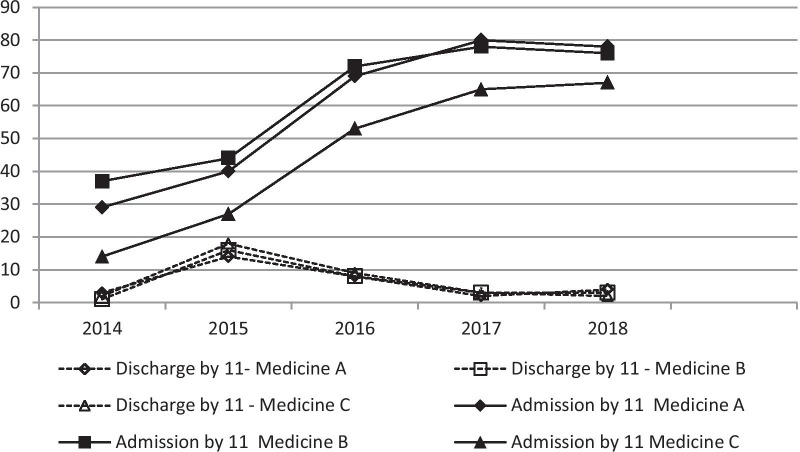
Morning discharges (%) by IM and admissions (%) from the ED, 2014–2018. *ED* emergency department. IM, internal medicine departments

The impact of the new admission intervention is shown in Fig. 2. As mentioned, the first stage (June 2014–January 2016) consisted of a major organizational effort in the medical departments to increase morning discharges. Prior to this study, IM department physicians would round on admitted patients in the morning, discharge patients after noon, and admit new patients in the afternoon and evening. Starting in June 2015, morning discharges increased from a baseline 4% (of all patients discharged during the 24-h day) to 18%. During the morning hours, a nurse supervisor would also circulate in the IM departments to assist the staff and trouble-shoot any logistic challenges possibly preventing timely discharges that day. However, cleaning the discharged patient beds and rooms, along with other demands on the nursing and medical staff would prove this increase in morning discharges unsustainable. With time, discharge rates decreased, prohibiting the desired transfer of 15–20 patients every morning to the IM departments. Accordingly, the second stage was initiated.

**Fig. 3:**
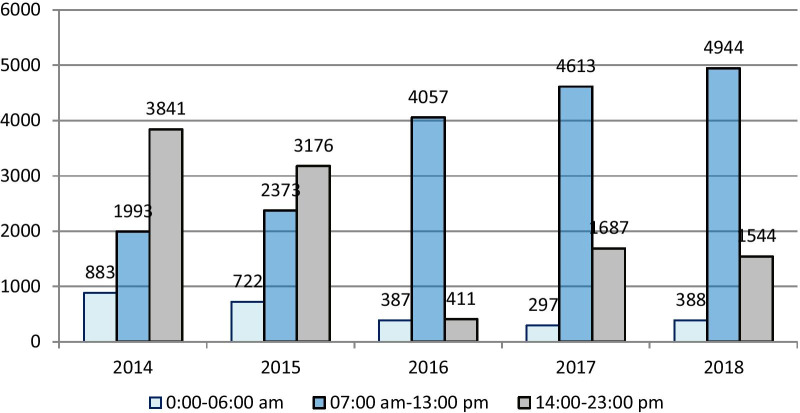
Admissions to IM departments, by hour of admission, n (%), 2014–2018. *IM* internal medicine departments

The second stage was initiated at the beginning of 2016. The adoption of the admission formula, as outlined earlier, led to a mean (± SD) of 35 ± 7 patients per day moving to the inpatient wards, averaging 8–12 patients per day per department. These patients were transferred to the IM departments regardless of bed availability. The percent of all patients being admitted to each medical department in the morning hours increased from a baseline of 30% (of all admissions from the ED) to 70% (Fig. 2). The 11:00 am time target for a significant number of discharges to occur was abandoned as there lacked sufficient numbers of physicians for rounding, discharging, and admitting new patients. The nurse-to-patient ratio also decreased (i.e., the number of patients per nurse increased) during this time. The number of patients present in each department at 7:00 am increased from a previous steady state of 36–38 to 42–44 patients. Peak daily number of patients became 45–50. With the increased admission frequency and volume, the pursuit of the admission target was achieved less consistently with a mean (± SD) of 4.4 ± 1 patients reaching each department by 2:00 pm, still constituting more than 70% of each department daily admissions from the ED (Fig. 3). During the intervention, there was no observed change in patient LOS.

## Discussion

The US Institute of Medicine has identified ED crowding as a critical threat to public health [10]. Exposure to over-crowded conditions in the ED has been shown to be associated with delays in care, decreased patient satisfaction, increased hospital LOS, and increased short-term mortality. Various strategies have been tried to improve throughput including ambulance diversion [11], a nurse navigator [12], a high turnover unit [13], and hold-over admissions [14, 15]—all of which are currently being assessed at our hospital. Nonetheless, ED boarding is endemic and increasing in our institution and elsewhere throughout the western world.

In order to control the amount of money that the HMOs spend on the purchase of hospital services (such as hospitalizations, procedures, etc.) and in order to prevent a dramatic increase in hospital services utilization, Israel created an economic mechanism more than two decades ago aimed at managing this process. The mechanism requires hospitals (service providers) to give discounts to the HMOs (service buyers) when the cost of services purchased (i.e., the HMOs) exceeds the amount they spent in a previous period. Typically, the comparison point is the average amount of service costs in the preceding three years. It should be noted that this mechanism may create a lack of incentive for the HMOs to prevent hospitalizations or create alternative services in the community.

These measures function via financial “incentives” when applied to the treatment and hospitalization of patients; specifically, administering significant fines on hospitals for the increase in healthcare expenditures compared to the previous year’s spending [16]. However, not like other admissions or procedures, HMOs receive no discount for referrals to the ED. This policy discourages referrals, and also aids in creating an impetus to develop community—based medical services and infrastructure (e.g., community emergency centers, call centers, and tele-medicine capabilities) to try and limit ED referrals. In contrast to the ER reimbursement policy, HMOs do receive a significant (up to 80%) discount for patients that are admitted to IM in cases where there is an increase in hospitalizations in the IM department compared to previous years [16]. This annual offset mechanism attempts to bridge financial gaps from year to year, as well as increase efficiency in the HMO's. However, from the perspective of the hospital, patient visits in the ED are profitable, whereas admission to IM are a money losing venture.

In summary, the reimbursement and pricing mechanism for visits to EDs and IM departments is significantly affected by the amount of discounts provided by the present law as described. In light of this, these assumptions make hospitalizations in the IM departments losses to hospitals, while visits to the emergency rooms are not money loosing ventures; especially, in view of the fact that the ED is the entrance gate to the hospital and an important source of its overall activity.

This paper describes a three-year experiment with a QI intervention to increase outflow of medical patients from the ED to the departments before noon, as 11:00 am was identified to be the time of day when the input of patients in the ED increases. The secondary purpose of the QI intervention was to increase IM discharge rate in the mornings to accommodate the surge in ED admissions. Unlike the United States, there is no incentive to discharge patients from the IM departments in Israel. In fact, on some days, the IM departments actually receive as many, or more, admissions than the number of patients discharged. While it was thought that resultant hallway admissions and crowding could be an “incentive” to increase patient discharge rate, this was not the case. Admitting a fixed number of patients to the IM departments regardless of bed availability was eventually associated with a decreased rate of discharge. This was probably multifactorial, to include lack of true incentive, observed overall increase of admissions to the ED over time, a intervention that effectively obviated the need for communication between the receiving IM department and ED, and system fatigue.

**Fig. 4:**
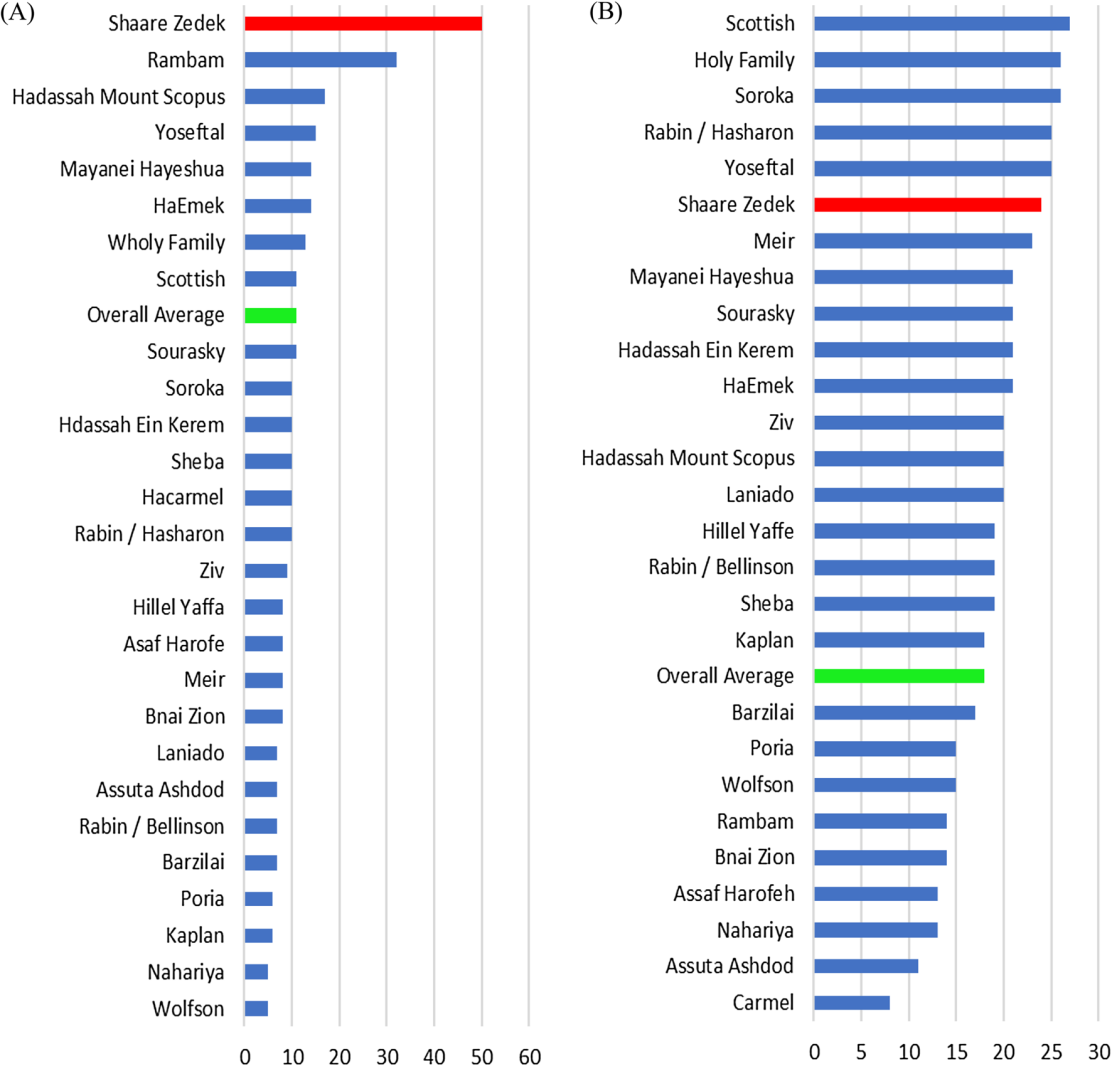
Percent of admissions to Internal Medicine by Israeli hospital and time of day, 2017. **A** 8:00 am to 12:00 pm, and **B** 12:00 pm to 4:00 pm

The chief beneficiary of this QI intervention, the ED, reported mild relief in the morning hours, only to return to pre-study levels of crowding in the evening. However, early in the study period, it appeared that the IM departments were unable to absorb the necessary number of patients determined by the prescribed formula. Consistent with our findings, the Israeli Ministry of Health published a report on emergency department admissions during 2017 (overlapping our study) (Fig. 4) [17]. SZMC demonstrated the greatest percentage of morning admissions from the ED to IM. In addition, this figure reflects the effect of ED overcrowding at other Israeli hospitals, not attempting the SZMC intervention. Even though peak admissions to the ED are during the evening hours, most Israeli hospitals admit patients to the hospital departments 24 h a day. The message to glean from this report is that the challenges we are witnessing with the present system are systemic. Over time, the number of patients getting admitted to IM and leaving the ED in the morning hours decreased. The median IM length of stay was unchanged during the study period. However, outlier patients remained in the hospital for a significantly longer period of time. This observation speaks to the more flexible discharge culture of our hospital, the lack of appropriate placement options and beds in the community, and the impact of significantly exceeding department occupancy rates.

The data show that the main impact for the IM departments was an increase in number of patients taken care of by the department teams, from a daily mean of 36–38 patients to 42–44. Furthermore, about 80% of all admissions to each medical department were in the morning hours, in addition to which each department received transfer patients from other departments. The medical or nursing teams did not increase in size, so the workload per staff member increased. Physicians responded by breaking up their teams. Instead of rounding together, allowing for discussion and teaching, each resident and intern was assigned a number of patients, with the attending physician overseeing their work. This fragmented care came at a cost of meaningful clinical interaction and learning opportunity, and increased dissatisfaction among physicians at all levels of seniority. The situation for the nurses was more complicated as the number of patients increased during the study to a peak ratio that strained the ability to deliver consistent high-quality care. In addition to the growing number of seriously ill patients per nurse, there was a dramatic increase in time each nurse has to spend on computer data entry (as result of MOH and Joint Commission accreditation requirements), leaving less time for patient interaction, leading to increased dissatisfaction.

Emergency department boarding of patients waiting for an IM hospital bed also had an adverse impact on the IM departments. When ED personnel have a limited ability to transfer patients to the departments, naturally, they will elect to transfer the sickest, most elderly, and debilitated patients. This biased selection process leaves the youngest and least ill in the ED, of whom a significant proportion will be discharged within 24–48 h. The result is a gravitational change in patient case-mix in the IM departments with a narrowing of spectrum of clinical challenges and an increase in mean age and mortality. The associated significant decrease in teaching opportunities for residents and students can make IM less attractive for potential residents.

**Fig. 5:**
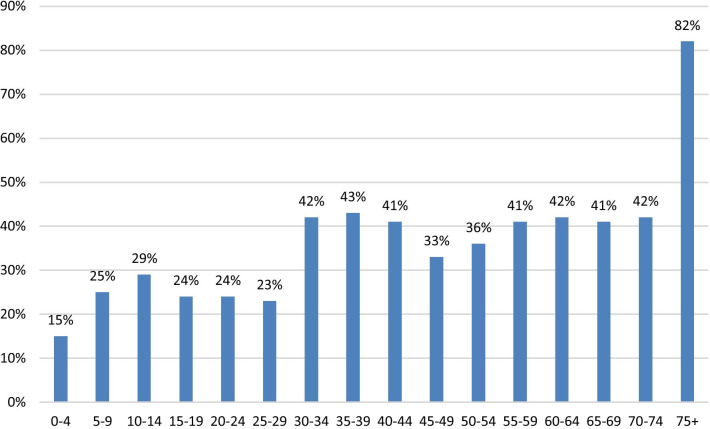
Predicted population growth in Jerusalem by age group, 2020–2035*. *Data reproduced with permission from the Jerusalem Institute for Policy Research, Jerusalem, Israel

The number of beds in long-term care facilities in Israel has not increased with demand and has actually decreased in Jerusalem. This has led to the accumulation of patients in IM departments waiting for placement. This explains the vast difference between relatively short median LOS and significantly longer mean standard deviation LOS for some patients, as each department houses several patients waiting several weeks or more for an available bed in a long-term care facility. In addition, SZMC is burdened with the largest aged population in country [18]. Not unexpected, hospitalization LOS at SZMC is longer than most of Israeli hospitals. Jerusalem is also home to the largest aged population in Israel and is expected to be so for years to come (Fig. 5). These demographics coupled with the significant lack of community healthcare beds only compound the problem of overcapacity within the hospital. However, this is not a local phenomenon, and these observations should direct healthcare policy globally.

Weaknesses of this paper include its single center and retrospective design. As such, there may have been unmeasured confounders. Patient safety events were not captured, and the impact of the QI intervention on patient satisfaction, a more qualitative metric, was not the focus of this paper. Patient-flow strategies, with characteristic similar to our intervention, have been attempted elsewhere in the world. For example, Kibubarajan et al. [19] conductive a large retrospective multicenter study of IM patients in Canada looking at morning discharges and associations with patient length of stay in inpatient IM departments. They found that the number of morning discharges were not significantly associate with shorter ED or IM LOS. They suggest that increasing the number of morning discharges alone is unlikely to substantially improve patient throughput in IM.

The limitations of this admission QI intervention have been delineated in this paper. In spite of initial expectations, the intervention did not contribute to a very significant outflow of patients from the ED, in particular in the morning hours. The initially larger number of patients leaving the ED gradually decreased IM bed availability. IM departments reached a new steady state, taking care of a mean of 42–44 patients (peaking at the middle of each day at 48–50) rather than 36–38. It remains to be determined whether the larger number of patients per staff member leads to decreased efficacy, although some observations raise concerns about quality of care and patient safety. In addition, SZMC started to actively solicit patient experience feedback only since 2021. Therefore, we were unable to assess patient satisfaction during the study. The concerns that the intervention did not realize its major goals, in addition to physician and nurse dissatisfaction, led to a set of corrective measures. First, the admission formula was changed to reduce the number of morning admissions and increase the number of afternoon and evening admissions. Second, IM Department D was moved from its site next to the ED to a full-size department next to the other three medical departments. Finally, a larger ED is being planned along with the addition of a full-size short-admission department. Hospital administrators need to demand system changes, some of which are suggested as policy targets in Table 2, that can only be implemented by the MOH [20].

**Table 2:** Policy targets to reduce patient crowding in ED and IM departments

1	Promotion of nursing home personnel to treat common clinical conditions that currently require transfer to hospitals
2	Increase bed availability in long-term care facilities
3	Increase hospitalization-at-home programs, both for the low-risk patients in the ED, and chronically ill IM patients awaiting a long-term care facility bed
4	Increase reimbursement for IM beds
5	Adjust bed numbers to mirror the increase of aged and dependent patients
6	Consider co-payment for patients, proxies, or nursing homes for unreasonable delays in discharge or demands for unsupported medical interventions (e.g., mechanical ventilation for the terminally ill or percutaneous endoscopic gastrostomy tube to prevent aspiration)
7	Utilize diagnostic related group-based reimbursement for IM admissions

*ED* emergency department, *IM* internal medicine departments

In conclusion, we describe a QI intervention in an attempt to improve outflow of medical patients from the ED in the morning hours. Unfortunately, the innovation resulted in a short-lived improvement in patient flow, as well as a range of untoward side effects, primarily for the IM departments. Nonetheless, in the crowded ED and IM departments that exist in Israel, hospitals need to experiment with various patient-flow strategies with the aim and hope to create higher efficiency. We continue to experiment with internal policies to improve patient flow but hope that the MOH will increase efforts to improve incentives to create more IM beds in hospitals and the community, amidst a backdrop of increasing chronic diseases and an ever-aging population.

## Data Availability

Available upon request.

## References

[1] Israeli Ministry of Health – Authority for economic and strategic planning 1995–2016. Hebrew. Assessed 27 June 2019. https://www.health.gov.il/PublicationsFiles.Stat1995_2016.pdf.

[2] Israeli Ministry of Health – Division of Information: The Authority for Technology and Medical Infrastructure. Hospitals and admission units, 2015. Part A: trends in hospitalizations. Part B: trends in patient admissions (Hebrew). Jerusalem: Israeli Ministry of Health Publications; 2016.

[3] Wang H, Robinson RD, Bunch K (2014). The inaccuracy of determining overcrowding status by using the national ED overcrowding study tool. Am J Emerg Med.

[4] Wang H, Kline JA, Jackson BE (2017). The role of patient perception of crowding in the determination of real-time patient satisfaction at emergency department. Int J Qual Health Care.

[5] Lord K, Parwani V, Ulrich A (2018). Emergency department boarding and adverse hospitalization outcomes among patients admitted to a general medical service. Am J Emerg Med.

[6] Derose SF, Gabayan GZ, Chiu VY, Yiu SC, Sun BC (2014). Emergency department crowding predicts admission length-of-stay but not mortality in a large health system. Med Care.

[7] Schuur JD, Venkatesh AK (2012). The growing role of emergency departments in hospital admissions. N Engl J Med.

[8] Bernstein SL, Aronsky D, Duseja R, Epstein S, Handel D, Hwang U (2009). The effect of emergency department crowding on clinically oriented outcomes. Acad Emerg Med.

[9] Yinnon AM, Munter G, Friedmann R (2014). Shaare Zedek Medical Center's model of an integrated division of internal medicine. Harefuah.

[10] Hospital-Based Emergency Care: At the breaking point—Institute of Medicine. http://www.iom.edu/Reports/2006/Hospital-Based-Emergency-Care-at-the-Breaking-Point.aspx. Accessed 27 June 2019.

[11] Olshaker JS, Rathlev NK (2006). Emergency department overcrowding and ambulance diversion: the impact and potential solutions of extended boarding of admitted patients in the emergency department. J Emerg Med.

[12] Fulbrook P, Jessup M, Kinnear F (2017). Implementation and evaluation of a navigator role to improve emergency department throughput. Australas Emerg Nurs J.

[13] Chang AM, Cohen DJ, Lin A (2018). Hospital strategies for reducing emergency department crowding: a mixed-methods study. Ann Emerg Med.

[14] Bump GM, Zimmer SM, McNeil MA, Elnicki M (2013). Hold-over admissions: are they educational for residents?. J Gen Intern Med.

[15] Pilgrim R, Hilton JA, Carrier E (2010). Research priorities for administrative challenges of integrated networks of care. Acad Emerg Med.

[16] https://www.gov.il/he/departments/policies/2016_dec1871.

[17] Israeli Ministry of Health. Report on visits to the Emergency Department, 2017. Information division, medical technologies division, information, and research department. 2019; 57.

[18] Israeli Ministry of Health. Report on Internal Medicine admissions 2005–2019. Information department, division of medical technology, data, and research. 2020.

[19] Kibubarajan A, Shin S, Fralic M (2021). Morning discharges and patient length of stay in inpatient general internal medicine. J Hosp Med.

[20] Schwartz Y, Jarjoui A, Yinnon AM (2019). Mechanically ventilated patients in medical departments: a necessary evil, or a blessing in (bad) disguise. Invited commentary. Isr J Health Policy Res.

